# Intersecting Pathways of Inflammation, Oxidative Stress, and Atherogenesis in the Evaluation of CKD: Emerging Biomarkers PCSK9, EPHX2, AOPPs, and TBARSs

**DOI:** 10.3390/life15081287

**Published:** 2025-08-13

**Authors:** Mohamed-Zakaria Assani, Marius Bogdan Novac, Anda Lorena Dijmărescu, Alexandra-Ștefania Stroe-Ionescu, Mihail Virgil Boldeanu, Isabela Siloși, Lidia Boldeanu

**Affiliations:** 1 Doctoral School, University of Medicine and Pharmacy of Craiova, 200349 Craiova, Romania; mohamed.assani@umfcv.ro (M.-Z.A.); alexandra.stroe@yahoo.com (A.-Ș.S.-I.); 2 Department of Immunology, Faculty of Medicine, University of Medicine and Pharmacy of Craiova, 200349 Craiova, Romania; isabela_silosi@yahoo.com; 3 Department of Anesthesiology and Intensive Care, Faculty of Medicine, University of Medicine and Pharmacy of Craiova, 200349 Craiova, Romania; mariusnovac2005@yahoo.com; 4 Department of Obstetrics and Gynecology, Faculty of Medicine, University of Medicine and Pharmacy of Craiova, 200349 Craiova, Romania; 5 Department of Microbiology, Faculty of Medicine, University of Medicine and Pharmacy of Craiova, 200349 Craiova, Romania; lidia.boldeanu@umfcv.ro

**Keywords:** chronic kidney disease, inflammation, oxidative stress, biomarkers, atherosclerosis

## Abstract

Chronic kidney disease (CKD) is a multifactorial disorder increasingly recognized as a systemic condition marked by persistent inflammation, oxidative stress, dyslipidemia, and endothelial dysfunction. Diabetic nephropathy, a leading cause of CKD, amplifies cardiovascular risk through intertwined mechanisms beyond traditional risk factors. This review synthesizes current evidence on the interplay between inflammation, oxidative stress, and atherosclerosis in CKD, with a special focus on emerging molecular biomarkers—PCSK9, EPHX2, AOPPs, and TBARSs—and their integration with clinical indices. These markers illuminate pathophysiological networks underlying CKD progression and cardiovascular complications, offering novel insights into risk stratification, disease monitoring, and targeted therapy. By exploring molecular and clinical intersections, this review underscores the potential of a personalized, biomarker-driven approach to CKD management.

## 1. Introduction

Chronic kidney disease (CKD) represents a growing global health burden, affecting more than 10% of the adult population and contributing significantly to morbidity, mortality, and healthcare costs. Metabolic disturbances, cardiovascular complications, and systemic inflammation frequently accompany its clinical progression. Among the most prevalent and severe causes of CKD is diabetic nephropathy (DN), a microvascular complication of type 2 diabetes mellitus (T2DM), which accounts for nearly 30–40% of end-stage renal disease (ESRD) cases worldwide. In this context, the interplay between inflammation, atherosclerosis, and oxidative stress emerges as a central pathophysiological mechanism driving both renal and cardiovascular outcomes [1,2,3,4,5,6].

CKD is now recognized not just as a kidney disorder but as a systemic disease marked by ongoing low-grade inflammation, endothelial dysfunction, and disrupted lipid metabolism. These changes drive atherosclerotic processes even in the early stages of CKD, making cardiovascular disease the leading cause of death in this group. Importantly, the traditional risk factors (such as hypertension, hyperlipidemia, diabetes, and smoking) do not fully account for the high cardiovascular burden observed in CKD, highlighting the need for more nuanced diagnostic and prognostic tools [3,7,8,9].

CKD is now among the leading causes of death globally and is projected to become the fifth leading cause of years of life lost by 2040 [10]. Despite improvements in the management of hypertension and diabetes—the most common causes of CKD—disease progression remains unpredictable in many patients [11]. This variability highlights the need for better biomarkers and a deeper understanding of the mechanisms that drive renal decline and associated complications.

CKD poses a rapidly escalating global public health challenge. According to the latest Global Burden of Disease data, CKD affected over 850 million people worldwide in 2021—an increase of nearly two-thirds in prevalence since 1990—with a projected age-standardized mortality rate rising from ~19.6 to over 21 per 100,000 by 2032 [12]. Regionally, the greatest growth in both incidence and burden has been observed in low- and middle-sociodemographic-index regions, with two-thirds of global cases residing in India and China [13]. Not only is CKD widespread, but its morbidity and mortality burden continue to increase. Between 1990 and 2019, CKD prevalence rose from approximately 6.7% to over 10%, and mortality rates surged accordingly [14].

Cardiovascular disease remains the predominant cause of death among CKD patients. A landmark meta-analysis including nearly 1.37 million individuals with non-dialysis CKD found a robust, independent increase in both all-cause and cardiovascular mortality risk, even after adjusting for traditional risk factors [15]. Further supporting the magnitude of this risk, a nationwide Danish registry study of over 138,500 patients with advanced CKD (eGFR < 30 mL/min/1.73 m^2^) reported a standardized 1-year cardiovascular mortality of ~9.8% in those with diabetes and ~7.4% without, compared to ~3.1% in matched controls from the general population [16].

One of the most notable features of CKD is its strong link to cardiovascular disease (CVD), which causes over 50% of deaths in affected individuals. The risk of cardiovascular issues in CKD is unusually high—even in those with only mild decreases in glomerular filtration rate—pointing to shared underlying processes beyond traditional risk factors [17]. Increasingly, attention has turned to the roles of inflammation, oxidative stress, and lipid-related endothelial dysfunction in mediating both kidney and cardiovascular injury [18].

Uremic toxins, metabolic derangements, and immune dysregulation fuel inflammation and oxidative stress in CKD. These pathways drive vascular calcification, glomerular sclerosis, and tubulointerstitial fibrosis, ultimately leading to progressive nephron loss [19]. Concurrently, lipid metabolism becomes distorted, not only in terms of serum lipid profiles but also through intracellular signaling molecules that regulate endothelial and immune cell behavior. These overlapping mechanisms form the basis of the so-called “cardiorenal syndrome,” in which vascular and renal injuries potentiate each other in a vicious cycle [20]. Notably, the pathophysiological landscape of CKD overlaps substantially with that of metabolic syndrome (MetS), as insulin resistance, dyslipidemia, and hypertension not only drive MetS progression but also accelerate renal injury and cardiovascular complications [21]. MetS serves as a prototype that engages complex pathogenic pathways, with insulin resistance (IR) identified as the predominant mechanism driving inflammation and atherosclerosis associated with CKD. The pathophysiology of MetS encompasses critical factors such as oxidative stress, persistent inflammation, endothelial dysfunction, and dysregulation of lipid metabolism [22,23,24].

As malnutrition, systemic inflammation, and cardiovascular disease are established determinants of poor outcomes in CKD, the prognostic nutritional index (PNI)—derived from serum albumin and lymphocyte count—has emerged as a valuable integrative marker of nutritional and immune status. Initially developed in oncology, the PNI has since been validated as a prognostic tool across diverse conditions including heart failure and COVID-19, where metabolic risk factors such as diabetes and obesity worsen outcomes. In geriatric CKD populations, a low PNI independently predicts higher mortality, highlighting its potential utility in risk stratification and guiding clinical management [25,26,27,28,29,30,31,32].

In one of our recent clinical studies [33], we have emphasized the value of integrating clinical indices, such as the atherogenic coefficient (AC) and the prognostic nutritional index (PNI), with metabolic and inflammatory markers to improve risk assessment in CKD patients. These indices, which reflect dyslipidemia and nutritional/inflammatory status, may serve as surrogate markers of cardiovascular and renal risk, particularly in individuals with T2DM and prediabetes.

Parallel to this, there is a growing focus on novel molecular biomarkers that potentially complement conventional indices, providing more granular insights into the pathophysiological mechanisms underpinning disease progression. Notable among these are proprotein convertase subtilisin/kexin type 9 (PCSK9), soluble epoxide hydrolase (EPHX2), advanced oxidation protein products (AOPPs), and thiobarbituric acid reactive substances (TBARS), which have demonstrated promise as biomarkers. These indicators are indicative of key pathogenic pathways such as lipid dysregulation, vascular inflammation, and oxidative stress, all of which are integral to the development and progression of CKD and its associated complications [34,35,36,37,38].

**Figure 1:**
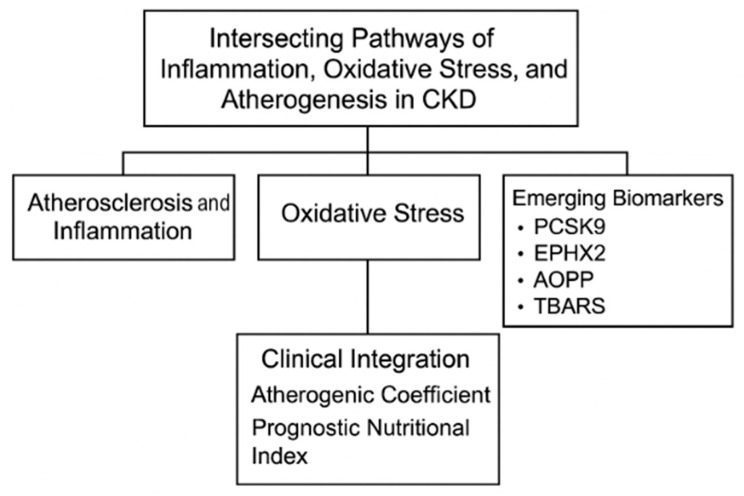
*The interplay of inflammation, oxidative stress and atherosclerosis.* (Figure created in BioRender) CKD: Chronic kidney disease; PCSK9: Proprotein convertase subtilisin/kexin type 9; EPHX2: soluble epoxide hydrolase; AOPPs: advanced oxidation protein products; TBARS: thiobarbituric acid reactive substances.

This review synthesizes the current evidence outlining the interconnected pathophysiological roles of inflammation, oxidative stress, and atherosclerosis in CKD progression, with an emphasis on emerging biomarkers such as PCSK9, EPHX2, AOPPs, and TBARSs, and their integration with established clinical indices like the AC and PNI, as depicted in Figure 1. A deeper understanding of these mechanistic links could facilitate the development of more precise, personalized diagnostic and therapeutic approaches in CKD management.

## 2. Atherosclerosis and Inflammation in Chronic Kidney Disease

### 2.1. Overview: A Vicious Cycle

Atherosclerosis and inflammation are intricately linked to the pathogenesis and progression of CKD. CKD patients exhibit accelerated vascular aging, characterized by arterial stiffness, endothelial dysfunction, and early-onset atherosclerosis [39,40,41]. Importantly, traditional cardiovascular risk factors—such as hypertension, diabetes, and dyslipidemia—do not fully explain the excessive cardiovascular morbidity and mortality observed in CKD patients, suggesting the presence of non-traditional, disease-specific mechanisms [42,43,44].

Low-grade chronic inflammation is now recognized as a hallmark of CKD and a driver of atherosclerosis. Proinflammatory cytokines (e.g., tumor necrosis factor-alpha (TNF-α); Interleukin 6 (IL-6); IL-1β) and acute-phase proteins such as C-reactive protein (CRP) are consistently elevated in CKD, promoting endothelial injury and smooth muscle proliferation [45,46]. Conversely, the uremic milieu in advanced CKD impairs endothelial nitric oxide production and exacerbates vascular calcification, creating a self-perpetuating inflammatory–atherogenic loop [47,48,49].

### 2.2. Inflammatory Mediators in CKD-Associated Atherosclerosis

Inflammation contributes to each stage of atherogenesis, from endothelial activation to plaque rupture. In CKD, impaired renal clearance leads to the accumulation of proinflammatory molecules such as advanced glycation end-products (AGEs), endotoxins, and indoxyl sulfate. These substances stimulate monocyte adhesion and migration, endothelial permeability, and foam cell formation, thus accelerating atheroma development [50,51,52].

IL-6 plays a central role by promoting hepatic synthesis of CRP and fibrinogen, enhancing thrombogenicity. Elevated CRP levels have been independently associated with cardiovascular events and mortality in dialysis and pre-dialysis populations [53,54]. However, CRP’s utility in CKD is limited by its lack of specificity, and it may be elevated due to dialysis-related inflammation as well [55]. In murine models, TNF-α, another key cytokine, induces insulin resistance, reduces endothelial nitric oxide synthase (eNOS) expression, and stimulates vascular adhesion molecule production, fostering monocyte recruitment to the endothelium [56,57].

Recent transcriptomic and proteomic studies have identified novel inflammatory signatures in CKD-associated atherosclerosis, including upregulation of Toll-like receptors (TLRs) such as Toll-like receptor 4 (TLR4), NOD-like receptor family, pyrin domain containing 3 (NLRP3) inflammasome components, and interferon regulatory factors IRF3 and IRF7 [58,59,60,61,62,63]. These insights may guide future therapeutic targeting of inflammation beyond broad-spectrum immunosuppressants. Metformin has been shown to inhibit NLRP3 inflammasome activation via AMPK-dependent mechanisms in kidney injury models, reducing IL-1β and TNF-α release and ameliorating renal inflammation in diabetic nephropathy and CKD contexts [64,65]. Curcumin, a natural polyphenol, directly suppresses NLRP3 inflammasome assembly and downregulates TLR4/myeloid differentiation primary response 88/nuclear factor-κB (TLR4/MyD88/NF-κB) pathways in CKD-related models, leading to decreased oxidative stress and inflammatory cytokine production [66]. Hydroxychloroquine inhibits endosomal TLR7 and TLR9 signaling, reducing type I interferon and TNF-α production in immune cells and attenuating macrophage activation and renal fibrosis in models of kidney injury [67].

### 2.3. Role of Dyslipidemia

PCSK9 is a serine protease mainly known for its role in controlling plasma LDL cholesterol levels by promoting LDL receptor degradation. In CKD, PCSK9 has gained interest not only because of its effect on dyslipidemia but also due to its involvement in inflammation. CKD is linked to increased circulating PCSK9 levels, which are believed to result from decreased kidney clearance. These higher levels are associated with endothelial dysfunction and heightened inflammatory signaling, including increased expression of vascular adhesion molecules and activation of proinflammatory cytokine pathways. Together, these effects contribute to the atherogenic environment characteristic of CKD, linking PCSK9 to both metabolic and vascular pathology in this population [34,68,69].

### 2.4. Endothelial Dysfunction and Vascular Calcification

CKD patients frequently exhibit endothelial dysfunction, characterized by reduced nitric oxide availability, oxidative stress, and increased expression of vascular adhesion molecules. Endothelial cells exposed to uremic toxins express high levels of E-selectin, Vascular Cell Adhesion Molecule-1 (VCAM-1), and Intercellular Cell Adhesion Molecule-1 (ICAM-1), promoting leukocyte infiltration and foam cell formation [7].

Moreover, vascular calcification—a common feature in CKD—is now understood to be an active, inflammation-driven process. Proinflammatory cytokines and oxidative stress promote vascular smooth muscle cell (VSMC) transdifferentiation into osteoblast-like cells, which deposit calcium phosphate in the arterial media [70,71]. This process not only stiffens vessels but also amplifies local inflammation, further enhancing atherogenesis.

**Figure 2:**
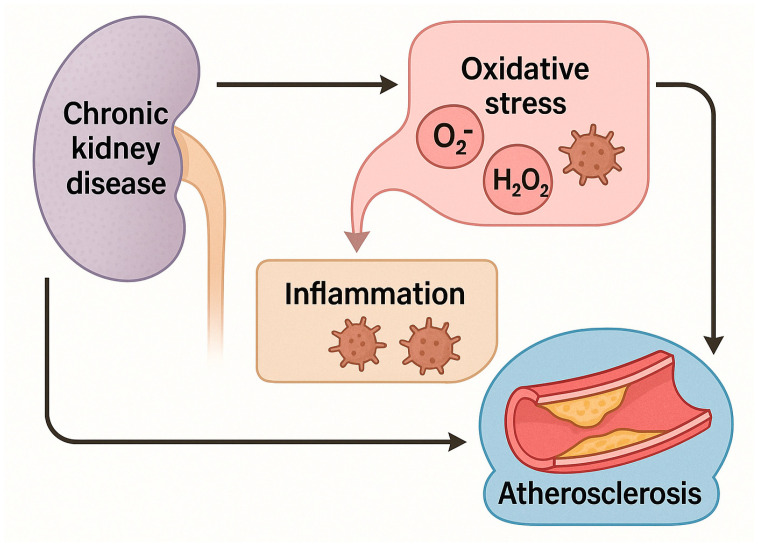
*Implications of atherosclerosis in chronic kidney disease (CKD).* (Figure created in BioRender) Schematic representation of the proposed pathophysiological links between CKD and atherosclerosis. CKD is associated with increased oxidative stress, characterized by elevated levels of reactive oxygen species (ROS), including superoxide (O_2_^−^) and hydrogen peroxide (H_2_O_2_). Oxidative stress promotes a pro-inflammatory state, and both oxidative stress and inflammation contribute to the initiation and progression of atherosclerosis. CKD may also exert direct pro-atherogenic effects through additional mechanisms not depicted. Arrows denote proposed causal relationships.

The EPHX2 enzyme, responsible for metabolizing epoxyeicosatrienoic acids (EETs), plays a pivotal role in endothelial dysfunction by attenuating vasodilatory and anti-inflammatory signaling pathways. Elevated EPHX2 activity has been implicated in CKD-related hypertension, albuminuria, and vascular inflammation [72,73]. Thus, EPHX2 represents another mechanistic bridge between inflammation and vascular injury in CKD. The implications of atherosclerosis are shown in Figure 2.

### 2.5. Inflammation as a Prognostic Indicator: PNI and Beyond

In addition to circulating cytokines, composite indices such as PNI have gained traction in CKD risk assessment. The PNI is calculated from the serum albumin and lymphocyte count—markers that reflect both nutritional status and immune function, as used in our recent studies [74]. In our study, T2DM patients with low PNI scores had elevated inflammatory markers, suggesting that malnutrition–inflammation syndromes are prevalent in diabetic nephropathy [33].

A low PNI has also been independently associated with all-cause mortality and cardiovascular events in hemodialysis patients [75]. Given its simplicity and dual relevance, the PNI may serve as a useful adjunct to traditional inflammatory biomarkers in CKD.

The interplay between inflammation, atherosclerosis, and CKD is intricate and bidirectional. While traditional biomarkers such as CRP and IL-6 continue to serve as valuable indicators, current evidence advocates for the incorporation of emerging inflammatory mediators such as PCSK9 and EPHX2 as well as novel indices like the AC or PNI into refined risk stratification frameworks. Nevertheless, the predominantly cross-sectional nature of existing research underscores the need for longitudinal studies to elucidate the prognostic significance of these biomarkers in CKD progression and cardiovascular morbidity.

## 3. Role of Oxidative Stress in Chronic Kidney Disease

Oxidative stress, characterized by an imbalance between the production of reactive oxygen species (ROS) and antioxidant defenses, is a critical factor in the pathogenesis and progression of CKD. The kidney is particularly susceptible to oxidative damage due to its high metabolic rate, dense vasculature, and reliance on mitochondrial energy production. In CKD, oxidative stress contributes not only to nephron injury and fibrosis but also exacerbates systemic complications, notably cardiovascular disease, anemia, and protein-energy wasting [76,77].

### 3.1. Mechanisms of Oxidative Stress in CKD

Several mechanisms underlie the heightened oxidative stress observed in CKD. These include the following [78,79,80]:

- Uremic toxin accumulation (e.g., indoxyl sulfate, p-cresyl sulfate), which induces mitochondrial dysfunction and NADPH oxidase activation (NOX);
- Inflammatory cytokines (e.g., IL-6, TNF-α), which amplify ROS production via activation of immune cells and endothelial cells;
- Impaired antioxidant systems, including reduced levels of glutathione, superoxide dismutase (SOD), catalase, and selenium-dependent enzymes.

Mitochondrial impairment is a critical contributor to oxidative stress in CKD pathophysiology. Dysfunctional mitochondria exhibit increased production of reactive oxygen species, including superoxide anions, hydrogen peroxide, and hydroxyl radicals, which induce oxidative damage to lipids, proteins, and nucleic acids. This leads to cellular senescence, apoptosis, and fibrogenesis—hallmarks of progressive nephron loss [81,82].

Additionally, oxidative stress directly damages the glomerular filtration barrier. Oxidation of glomerular basement membrane proteins and podocyte actin filaments impairs filtration function, contributing to proteinuria and further renal decline [83,84].

Reduced glutathione (GSH) and SOD form pivotal components of the cellular antioxidant defense system. SOD catalyzes the conversion of superoxide radicals into hydrogen peroxide, which is then detoxified by GSH-dependent enzymes such as glutathione peroxidase. In CKD, studies consistently report decreased activity of SOD and lower GSH levels in plasma and cells of both non-dialysis and dialysis patients, indicating a compromised redox buffering capacity [80]. When these defenses are depleted, ROS accumulate unchecked, leading to oxidative damage across multiple cellular structures. Endothelial dysfunction is marked by decreased nitric oxide bioavailability and elevated lipid peroxidation. Chronic oxidative stress induces fibrogenic signaling in renal tubular epithelial cells, facilitating tubulointerstitial fibrosis and leading to a progressive decline in eGFR [80,85]. From a clinical perspective, decreased levels of antioxidant enzymes have been associated with increased proteinuria, accelerated CKD progression, and elevated rates of cardiovascular events [80,86].

### 3.2. Lipid Peroxidation and TBARSs

Lipid peroxidation is a major consequence of ROS activity and plays a pivotal role in both renal and vascular injury. ROS attack polyunsaturated fatty acids in cell membranes, generating reactive aldehydes such as malondialdehyde (MDA)and 4-hydroxynonenal (4-HNE). These compounds form adducts with DNA and proteins, amplifying cellular damage and inflammation [87,88]. One of the most widely used assays to assess lipid peroxidation is the measurement of TBARSs, which primarily reflects MDA levels. Elevated TBARS levels have been reported in all stages of CKD, including early-stage disease, dialysis patients, and renal transplant recipients [89]. High TBARS levels correlate with the following [90,91]:

- A reduced glomerular filtration rate (GFR);
- An increase in cardiovascular events;
- Endothelial dysfunction and arterial stiffness.

### 3.3. Protein Oxidation and AOPPs

While lipid peroxidation reflects membrane damage, protein oxidation mirrors structural and enzymatic dysfunction. One of the most specific markers in this domain is AOPPs—dityrosine-containing protein aggregates formed during oxidative modification of plasma proteins, especially albumin, by chlorinated oxidants (e.g., hypochlorous acid) [92,93].

AOPP levels are significantly elevated in patients with moderate-to-advanced CKD and are particularly high in individuals undergoing hemodialysis. Their levels are strongly associated with the following [89,94,95,96]:

- Inflammation (correlating with CRP and IL-8);
- Malnutrition (inverse relationship with albumin);
- Endothelial dysfunction and arterial stiffness.

Importantly, AOPPs are not just bystanders—they actively contribute to disease progression. AOPPs can stimulate monocyte activation, trigger NOX pathways, and promote vascular smooth muscle proliferation. They also induce tissue factor expression and enhance thrombogenic potential, linking oxidative stress to procoagulant states seen in CKD [19,97].

Furthermore, studies have shown that AOPPs upregulate the expression of transforming growth factor-beta (TGF-β) and collagen IV in tubular cells, suggesting a direct role in renal fibrosis [98,99]. This expands their relevance from biomarker to potential therapeutic target.

### 3.4. Other Key Redox Pathways: Nrf2/Keap1 Signaling, Mitochondrial ROS Regulation, MitoQ, Bardoxolone Methyl

The nuclear factor erythroid 2–related factor 2 (Nrf2) pathway, regulated by its inhibitor Keap1, is a master transcriptional regulator of cellular antioxidant defenses. In CKD models, Nrf2 activity is suppressed, leading to reduced expression of heme o1xygenase-1 (HO-1), SOD, and NADPH:quinone oxidoreductase 1(NQO1), and contributing to oxidative injury, inflammation, and renal fibrosis. Clinical and preclinical data highlight impaired Nrf2 activation in diabetic nephropathy and remnant kidney models [100,101,102,103].

Mitochondria are a principal source of reactive oxygen species, particularly via complexes I and III of the electron transport chain. CKD is associated with mitochondrial dysfunction—impaired mitophagy, compromised biogenesis, and disrupted respiratory chain integrity—that amplifies ROS production and drives tubular apoptosis, podocyte injury, and endothelial dysfunction [103,104,105].

Mitochondria-targeted antioxidant (Mitochondria-targeted Quinone (MitoQ); Skulachev Quinone 1 (SkQ1)) agents accumulate within mitochondria to neutralize ROS at the source. Preclinical models show that MitoQ and related compounds reduce oxidative damage, improve endothelial function, and attenuate proteinuria in CKD contexts. Human safety data suggest good tolerability without nephrotoxicity in acute dosing trials [105,106,107,108].

A synthetic triterpenoid that activates Nrf2 and suppresses NF-κB, bardoxolone methyl (CDDO-Me) significantly improved estimated GFR in early-phase CKD trials, including the BEAM and TSUBAKI studies—though the pivotal BEACON Phase III trial was halted due to increased cardiovascular events [109,110,111].

### 3.5. Interactions with Traditional Risk Factors and Comorbidities

Oxidative stress interacts with many conventional risk factors to amplify renal injury [89,112,113,114]:

- Diabetes mellitus: hyperglycemia induces ROS via AGEs and polyol pathway flux;
- Hypertension: angiotensin II stimulates ROS generation through activation of NOX and impairs endothelial nitric oxide synthesis;
- Dyslipidemia: oxidized LDL further promotes ROS production and immune activation.

The cumulative effect of these insults contributes to a chronic oxidative–inflammatory state that drives both glomerulosclerosis and atherogenesis. This pathophysiological overlap supports the rationale for studying shared biomarkers like TBARSs and AOPPs, which may reflect both renal and vascular stress. Oxidative stress is a cornerstone of CKD pathophysiology, driving both renal injury and systemic complications. Biomarkers such as TBARSs and AOPPs offer valuable insights into lipid and protein oxidation, respectively, and correlate with disease severity and prognosis. As evidence grows, these oxidative markers may serve not only as diagnostic tools but also as therapeutic targets in strategies aimed at slowing CKD progression and reducing cardiovascular events [80,115].

Moreover, elevated oxidative stress worsens anemia in CKD by shortening erythrocyte lifespan and impairing erythropoietin responsiveness, contributing to fatigue and cardiovascular risk [116,117].

In diabetic patients, persistent hyperglycemia drives the formation of AGEs. AGEs bind to their receptor (RAGE) on podocytes, endothelial cells, and mesangial cells, which activates NOX and triggers excessive ROS generation. This oxidative burst induces NF-κB–mediated inflammatory gene expression and promotes podocyte apoptosis and mesangial expansion—hallmarks of diabetic nephropathy progression [76,118].

### 3.6. Potential Therapeutic Targets and Antioxidant Strategies

Given its central role in CKD progression, oxidative stress has become a target for therapeutic intervention. Several antioxidant strategies have been evaluated with varying success [78,110,119,120,121]:

- Vitamin E and C supplementation has shown modest effects in reducing TBARS and improving endothelial function, particularly in early-stage CKD;
- N-acetylcysteine (NAC) replenishes glutathione stores and has been shown to reduce proteinuria and improve oxidative markers in some trials;
- Bardoxolone methyl is an Nrf2 activator that enhances endogenous antioxidant defenses; while promising in phase II trials, it showed adverse cardiovascular outcomes in later-stage CKD.

Emerging agents such as mitochondria-targeted antioxidants (e.g., MitoQ, SkQ1) and EPHX2 inhibitors are being investigated for their dual antioxidant and anti-inflammatory properties. These agents may represent a more targeted approach to mitigate ROS generation at its source [72,107,122,123]. Oxidative stress is central to CKD pathophysiology, yet the clinical utility of antioxidant supplementation remains unproven. A comprehensive Cochrane review assessed randomized controlled trials of various antioxidants—such as vitamin E, C, N-acetylcysteine, and others—in adults with CKD across all stages, including dialysis. The review reported that the antioxidant mentioned showed modest effects on oxidative markers and proteinuria but did not consistently improve survival or slow CKD progression [124].

## 4. Integrated Roles of PCSK9, EPHX2, AOPPs, and TBARSs in CKD Pathophysiology

The pathogenesis of CKD is multifactorial but increasingly understood to involve a synergistic network of dysregulated lipid metabolism, oxidative stress, and chronic inflammation. In this context, the biomarkers PCSK9, EPHX2, AOPPs, and TBARSs might reflect distinct yet overlapping pathways that drive renal injury, atherogenesis, and cardiovascular complications [34,125,126].

PCSK9 primarily regulates LDL receptor turnover but also participates in vascular inflammation and foam cell formation. Elevated PCSK9 levels are linked to worsening eGFR, albuminuria, and cardiovascular risk in CKD patients—even independent of lipid profiles. Its proinflammatory actions through TLR4 and NF-κB signaling establish it as a dual lipid–inflammatory marker, bridging metabolic and vascular injury [127,128,129,130].

EPHX2 encodes soluble epoxide hydrolase, which degrades vasoprotective EETs, promoting vasoconstriction, oxidative stress, and endothelial dysfunction. Its overexpression in diabetic and hypertensive nephropathies correlates with proteinuria and renal fibrosis. Unlike PCSK9, which acts systemically, EPHX2 plays a renal-localized role, particularly in the tubulointerstitium and microvasculature [5,131,132].

AOPPs represent oxidatively modified plasma proteins—primarily albumin—formed by neutrophil-derived oxidants. They are not passive byproducts but bioactive DAMPs that activate NOX, RAGE, and proinflammatory cytokines. Elevated AOPP levels are associated with glomerulosclerosis, malnutrition–inflammation syndrome, and vascular stiffness in both dialysis and non-dialysis CKD patients [85,133,134,135].

TBARSs, mainly reflecting MDA, are indicators of lipid peroxidation and systemic oxidative stress. Their elevation correlates with dyslipidemia, endothelial dysfunction, and cardiovascular events. Although less specific than AOPPs, TBARSs are particularly useful for tracking oxidized lipoprotein burden and lipid membrane injury, both key in atherogenesis [77,136,137,138,139].

A comparative summary is displayed in Table 1:

### 4.1. PCSK9: A Link Between Dyslipidemia, Inflammation, and CKD Progression

#### 4.1.1. Overview

PCSK9 is a circulating serine protease primarily expressed in the liver. It plays a pivotal role in cholesterol metabolism by promoting the lysosomal degradation of low-density lipoprotein receptors (LDLR) on hepatocytes, thereby increasing plasma LDL cholesterol (LDL-C) levels. Beyond its well-established role in lipid homeostasis, PCSK9 is increasingly recognized as a pleiotropic molecule involved in vascular inflammation, endothelial dysfunction, and renal disease progression, making it a promising biomarker and therapeutic target in CKD and its cardiovascular complications [140,141,142,143].

A 2025 review article illuminates a novel mechanism whereby PCSK9 interacts with megalin, a proximal-tubule protein vital for protein reabsorption. PCSK9-mediated megalin degradation leads to proteinuria; inhibition of PCSK9 in animal models preserved megalin, reduced proteinuria, and yielded renal benefit [144]. Beyond lipid regulation, PCSK9 directly influences tubular protein handling, linking it mechanistically to renal injury [144,145,146].

A recent prospective cohort study by Liu et al. [129], which followed 1902 individuals with type 2 diabetes over a median period of 7.2 years, demonstrated that elevated baseline plasma PCSK9 levels were independently linked to a higher risk of CKD progression, defined as either a doubling of serum creatinine or the onset of end-stage kidney disease. Each standard deviation increase in PCSK9 was associated with a 27% greater risk, while individuals in the highest tertile faced a 47% increased risk compared to those in the lowest tertile.

#### 4.1.2. Mechanisms of PCSK9 in Lipid and Inflammatory Pathways

In physiological states, LDL receptors bind circulating LDL particles and internalize them, removing atherogenic lipoproteins from the bloodstream. PCSK9 binds to LDLR and targets them for lysosomal degradation, limiting LDLR recycling and promoting LDL accumulation. Statins, while lowering cholesterol, paradoxically upregulate PCSK9 expression, which may attenuate their lipid-lowering effect—a phenomenon known as the “PCSK9 rebound” [147,148,149].

Beyond lipid regulation, PCSK9 exhibits proinflammatory actions. It enhances expression of IL-1β and TNF-α in monocytes and macrophages, promotes vascular smooth muscle proliferation, and increases oxidized LDL (oxLDL) uptake, fueling foam cell formation. PCSK9 has also been shown to activate TLR4 signaling pathways, linking it directly to innate immune responses and atherosclerosis [34,150,151,152,153,154].

These proatherogenic and immunomodulatory effects position PCSK9 as a potential mechanistic link between dyslipidemia, vascular inflammation, and kidney damage.

#### 4.1.3. PCSK9 in CKD and Diabetic Nephropathy

Several studies have demonstrated altered PCSK9 levels in CKD, with both overexpression and dysregulated clearance observed in patients with a reduced GFR. In early-stage CKD, plasma PCSK9 levels may increase due to compensatory hepatic overproduction. In advanced stages, accumulation may occur due to impaired renal clearance, mirroring the retention of other middle molecules [34,155,156,157].

In patients with DN, elevated PCSK9 levels correlate positively with albuminuria, LDL-C, and CRP, and inversely with eGFR. These findings underscore PCSK9’s potential as a biomarker for glomerular injury and inflammation in diabetes-related kidney disease [158].

Moreover, PCSK9 expression has been detected in renal tubular epithelial cells, especially under hyperglycemic or proinflammatory conditions. This suggests a potential role in local renal pathophysiology beyond hepatic cholesterol handling [159,160].

#### 4.1.4. Association with Cardiovascular Risk in CKD

Recent studies have also linked PCSK9 levels with pulse wave velocity (PWV), carotid intima-media thickness (cIMT), and coronary artery calcification scores in CKD and dialysis cohorts, further validating its role in vascular remodeling and cardiovascular risk stratification [161,162,163,164].

Emerging evidence indicates that elevated PCSK9 levels are independently associated with an increased risk of MACEs, particularly among women, and with all-cause mortality, particularly in men. Additionally, PCSK9 concentrations have been shown to correlate with markers of impaired glucose homeostasis and an atherogenic lipid profile, further underscoring its role in cardiometabolic risk stratification [131,165].

#### 4.1.5. PCSK9 Inhibitors: Therapeutic Relevance in CKD

Two classes of PCSK9-targeted therapies have entered clinical practice [166]:

- Monoclonal antibodies (e.g., evolocumab, alirocumab), which bind circulating PCSK9, preventing it from degrading LDL receptors;
- siRNA-based therapies (e.g., inclisiran) which silence PCSK9 mRNA in hepatocytes, offering sustained inhibition with infrequent dosing.

These agents significantly reduce LDL-C by up to 60%, and clinical trials (e.g., FOURIER, ODYSSEY OUTCOMES) have demonstrated reductions in MACEs, even in high-risk populations [167,168]. The FOURIER cardiovascular outcome trial included 4443 participants with stage ≥3 CKD and demonstrated that evolocumab lowered LDL-C by approximately 58–59% across CKD strata and reduced the risk of major cardiovascular events similarly in patients with preserved, stage 2, or stage ≥3 kidney function [169,170,171]. The absolute reduction in cardiovascular risk over 30 months was numerically larger in those with more severe CKD (≈2.5% vs. 1.7%), reflecting higher baseline event rates [170]. Moreover, observational series and pooled analyses confirm that PCSK9 inhibitors are safe and effective in mild-to-moderate CKD, with LDL-C-driven benefits and preserved renal function, although data in eGFR <30 are still limited [172,173].

Beyond lipid lowering, PCSK9 inhibition may exert direct renoprotective effects. Clinical cohorts report reductions in albuminuria and proteinuria among CKD patients treated with PCSK9 inhibitors [174]. Experimental work shows that blocking PCSK9 preserves proximal tubular megalin expression, improving protein reabsorption and mitigating proteinuria in diabetic and nephrotic models [175].

Additionally, early data suggest that PCSK9 inhibition may exert anti-inflammatory effects, reducing high-sensitive C-reactive protein (hs-CRP) and monocyte activation markers. This opens the door to pleiotropic benefits in CKD beyond lipid control [176].

PCSK9 plays a multifaceted role in CKD, contributing to lipid dysregulation, vascular inflammation, and potentially direct renal injury. Elevated levels are associated with worse renal function, higher cardiovascular risk, and increased inflammation. While PCSK9 inhibitors offer a promising therapeutic avenue, their role in advanced CKD and dialysis populations requires further study [144,170]. A 2025 study by Hummelgaard et al. evaluated the long-term effects of dual inhibition of PCSK9 (using alirocumab) and SGLT2 (using empagliflozin) in obese ZSF1 rats, a translational model of diabetic nephropathy and metabolic-syndrome–associated CKD. Contrary to expectations, neither alirocumab nor empagliflozin—alone or combined—conferred renoprotective benefits after 15 months: there was no attenuation of proteinuria, no preservation of eGFR surrogate measures, and no reduction in renal histopathological injury (glomerulosclerosis, interstitial fibrosis) compared to the controls [177].

Statin treatment significantly confounds the interpretation of circulating PCSK9 levels. Statins upregulate PCSK9 transcription via activation of sterol regulatory element-binding proteins (SREBP-2), leading to increased circulating PCSK9 even as LDL-C falls [149,178]. In CKD cohorts, particularly those on maintenance hemodialysis, studies such as the GCKD cohort have demonstrated that statin use was the strongest independent predictor of elevated PCSK9, with mean serum PCSK9 levels being approximately 70 ng/mL higher in statin-treated individuals, while kidney function markers like eGFR showed no independent association with PCSK9 levels [131].

Integrating PCSK9 with clinical indices and other biomarkers could enhance personalized medicine approaches to CKD management, particularly in patients with high residual cardiovascular risk despite standard therapies.

### 4.2. EPHX2: A Mediator of Vascular Inflammation and Renal Injury

#### 4.2.1. Overview

Soluble epoxide hydrolase, encoded by the EPHX2 gene, is an enzyme that hydrolyzes epoxyeicosatrienoic acids (EETs) into their less active dihydroxyeicosatrienoic acid (DHET) forms. EETs are lipid signaling molecules derived from arachidonic acid via cytochrome P450 epoxygenases. They exert beneficial effects on the cardiovascular and renal systems by promoting vasodilation, anti-inflammation, antithrombosis, and cytoprotection [72,179,180].

By degrading EETs, EPHX2 diminishes their protective effects, the balance toward vasoconstriction, inflammation, and fibrosis. Recent studies highlight the role of EPHX2 as a crucial mediator of endothelial dysfunction, glomerular injury, and progressive CKD. EPHX2 is thus emerging as a promising biomarker and drug target for renal and cardiovascular diseases, particularly in populations characterized by oxidative stress and systemic inflammation [72,181,182].

#### 4.2.2. Mechanistic Role of EPHX2 in Vascular and Renal Pathophysiology

Under physiological conditions, EETs produced by endothelial cells act as endothelium-derived hyperpolarizing factors (EDHFs), enhancing vasodilation through calcium-activated potassium channels. EETs also inhibit NF-κB signaling, reducing the expression of proinflammatory cytokines, adhesion molecules, and ROS [183,184].

EPHX2-mediated degradation of EETs disrupts these pathways, leading to the following [72,185,186]:

- Increased vascular tone and hypertension;
- Enhanced monocyte adhesion and infiltration;
- Elevated oxidative stress;
- Proliferation of vascular smooth muscle cells;
- Renal tubular apoptosis and interstitial fibrosis.

**Figure 3:**
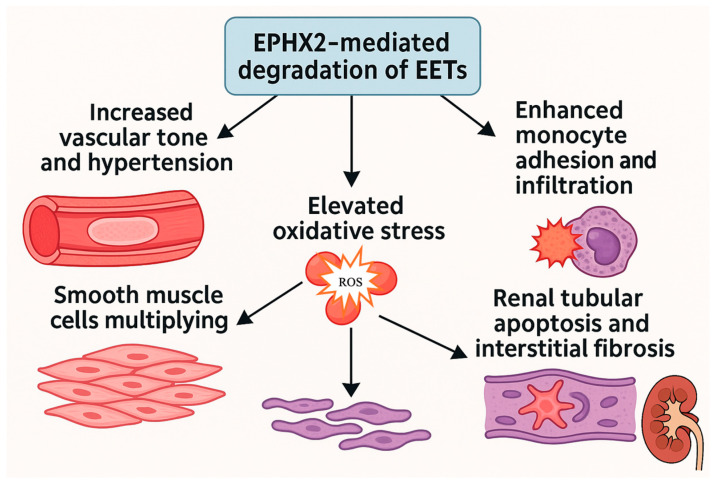
*Pathophysiological consequences of EPHX2-mediated degradation of EETs.* (Figure created in BioRender) This infographic illustrates how EPHX2-mediated degradation of epoxyeicosatrienoic acids (EETs) disrupts protective vascular and renal pathways, leading to multiple pathological outcomes. The enzyme-driven loss of EETs contributes to increased vascular tone and hypertension, enhanced monocyte adhesion and infiltration, elevated oxidative stress, proliferation of vascular smooth muscle cells, and renal tubular apoptosis with interstitial fibrosis. Together, these processes highlight the central role of EPHX2 activity in promoting vascular dysfunction and kidney injury.

Figure 3 summarizes the effects of EPHX2-mediated degradation of EETs.

These effects are particularly pronounced in the renal microvasculature, where soluble epoxide hydrolase (sEH) is highly expressed in proximal tubules, afferent arterioles, and glomerular endothelial cells. The overactivity of EPHX2 contributes to renal ischemia, proteinuria, and glomerulosclerosis, all hallmarks of progressive CKD [72,187,188].

In addition to renal effects, EPHX2 is heavily implicated in the cardiovascular complications of CKD. Elevated sEH activity contributes to the following [189,190,191,192]:

- Arterial stiffness and calcification;
- Endothelial dysfunction via suppression of EET-mediated nitric oxide signaling;
- Myocardial remodeling and left ventricular hypertrophy;
- Enhanced oxidative stress and vascular inflammation.

In CKD patients, where cardiovascular mortality is disproportionately high, these effects are magnified by uremia and dysregulated lipid metabolism. Given the anti-inflammatory and vasodilatory properties of EETs, restoring their levels via EPHX2 inhibition offers a dual benefit: slowing renal decline and reducing cardiovascular risk. EPHX2 represents a key mediator of vascular inflammation and renal injury in CKD. Through degradation of EETs, EPHX2 promotes endothelial dysfunction, oxidative stress, and fibrosis—mechanisms central to CKD progression and its cardiovascular complications [44,72].

A 2025 study analyzed DNA methylation patterns in kidney tissue from patients with diabetic kidney disease (DKD). They found specific methylation changes at CpG sites within the EPHX2 gene linked to gene expression changes and DKD severity. The authors suggest EPHX2 methylation levels could serve as novel epigenetic biomarkers for CKD progression in diabetes. Beyond enzyme activity, epigenetic regulation of EPHX2 may reflect or drive disease progression, offering new diagnostic angles [193].

#### 4.2.3. EPHX2 Inhibitors: Preclinical and Early Clinical Insights

While no large-scale trials have yet tested EPHX2 inhibitors in CKD patients, preclinical models provide compelling support: genetic deletion or pharmacological blockade of sEH attenuates renal inflammation and tubulointerstitial fibrosis in multiple CKD models (e.g., diabetic nephropathy, remnant kidney, high-fat diet) through enhanced EET bioavailability and modulation of oxidative stress and autophagy pathways [72,194]. A recent 2024 review highlights that early-phase clinical studies using EPHX2 inhibitors (such as GSK2256294) are underway in acute kidney injury and diabetic nephropathy, aimed at reversing oxidative injury and preserving tubular function [72]. Human studies reveal [126] that epigenetic modifications influence CKD progression. DNA methylation changes in the EPHX2 gene correlate with altered expression and disease severity in diabetic kidney disease, implicating epigenetic regulation of soluble epoxide hydrolase in renal inflammation and fibrosis. Furthermore, genetic association studies link EPHX2 variants to accelerated CKD progression, underscoring both genetic and epigenetic contributions [72].

### 4.3. AOPPs: A Marker and Mediator of Oxidative Protein Damage in CKD

#### 4.3.1. Overview

AOPPs are dityrosine-containing protein cross-linking products generated primarily by the reaction of plasma proteins—especially albumin—with chlorinated oxidants such as hypochlorous acid (HOCl) and chloramines, both released by activated neutrophils and monocytes via the myeloperoxidase (MPO) pathway. Originally described in 1996, AOPPs are now recognized as both biomarkers of oxidative stress and active contributors to the pathogenesis of CKD and its cardiovascular complications [92,195,196].

Unlike lipid peroxidation markers (e.g., TBARSs), AOPPs reflect protein oxidation, which can lead to irreversible changes in protein structure and function. Elevated AOPP levels indicate ongoing oxidative and inflammatory stress, correlating with renal dysfunction, malnutrition, vascular damage, and mortality risk in CKD patients [80,89,97].

#### 4.3.2. Formation and Biochemical Characteristics

AOPPs are primarily derived from the following [76,197]:

- Oxidized albumin and other plasma proteins;
- Interaction with MPO-derived oxidants, which accumulate due to immune activation and poor clearance in CKD.

These modified proteins contain cross-linked tyrosine residues and carbonyl groups, which resist proteolysis and can accumulate in tissues. In addition to acting as inert markers, AOPPs are bioactive and can activate inflammatory signaling, including the following [198]:

- NOX activation;
- NF-κB pathway stimulation;
- RAGE binding.

This proinflammatory signaling cascade contributes to vascular inflammation, endothelial dysfunction, and glomerular injury, forming a pathogenic loop that drives CKD progression [199].

#### 4.3.3. Inflammation and Immune Activation

AOPPs act as danger-associated molecular patterns (DAMPs), engaging pattern recognition receptors such as RAGE and TLR4 on immune and endothelial cells. This leads to increased production of the following [200]:

- Proinflammatory cytokines (e.g., TNF-α, IL-6);
- Endothelial adhesion molecules (VCAM-1, ICAM-1);
- ROS-generating enzymes (e.g., NOX4, iNOS).

These changes reinforce systemic inflammation, a well-known driver of vascular injury and atherosclerosis in CKD [89].

#### 4.3.4. Atherosclerosis and Vascular Calcification

AOPPs contribute to atherosclerosis in the following ways [201,202,203]:

- Promoting LDL oxidation, enhancing foam cell formation;
- Inducing vascular smooth muscle cell (VSMC) proliferation and osteogenic transdifferentiation;
- Inhibiting eNOS, impairing vasodilation.

**Figure 4:**
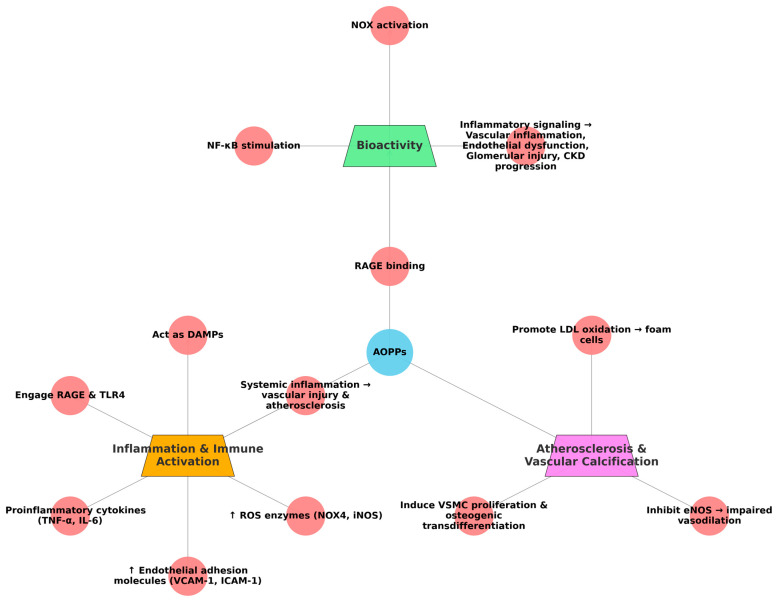
*Interplay of AOPPs with inflammation, atherosclerosis, and vascular dysfunction in CKD.* (Figure created in BioRender) The diagram illustrates how AOPPs trigger bioactive signaling, drive inflammation, and contribute to vascular and glomerular injury, highlighting their central role in CKD progression, atherosclerosis, and malnutrition. ↑ = activate.

For a holistic overview of AOPPs’ interplay in CKD, we have synthetized the processes in Figure 4.

AOPPs are robust markers of oxidative protein damage and active mediators of renal and vascular pathology in CKD. They contribute to systemic inflammation, atherosclerosis, and protein-energy wasting—all major contributors to poor outcomes in this population [84,89]. The comprehensive review by Tsinari et al. highlights that circulating AOPP levels rise progressively with advancing CKD stage and are markedly elevated in dialysis patients, where they correlate with increased arterial stiffness, malnutrition–inflammation scores, and adverse cardiovascular outcomes. The review also emphasizes that AOPPs behave not only as surrogate biomarkers of oxidative burden but also as causal drivers: they can activate NOX, induce endothelial dysfunction, and amplify inflammatory cytokine release, thus perpetuating a cycle of oxidative stress and tissue injury [204].

### 4.4. TBARSs: A Classical Marker of Lipid Peroxidation in CKD

#### 4.4.1. Overview

TBARSs are a group of compounds that react with thiobarbituric acid (TBA) to form chromogenic adducts, most notably MDA—a well-established marker of lipid peroxidation. TBARS measurement remains one of the most widely used, albeit nonspecific, assays to evaluate oxidative stress in both research and clinical settings [205,206].

A 2025 cross-sectional study showed significantly elevated MDA (TBARS) levels in both hemodialysis and peritoneal dialysis patients, strongly correlated with CRP (r = 0.68) and reduced antioxidant enzyme activity, underscoring TBARS as a robust oxidative–inflammatory biomarker in the dialysis setting. These data reinforce the use of TBARSs as a monitoring tool in dialysis, reflecting both oxidative injury and inflammatory status, and supporting their role in patient risk stratification [207].

In CKD, where oxidative stress plays a central role in pathogenesis and cardiovascular complications, TBARS levels are consistently elevated and correlate with disease severity, inflammation, and vascular damage. While not as specific as AOPPs for protein oxidation, TBARSs provide valuable insights into membrane lipid damage, lipoprotein oxidation, and oxidative–inflammatory loops that drive CKD progression [208,209,210].

#### 4.4.2. Biological Significance

MDA is generated from the oxidative degradation of polyunsaturated fatty acids (PUFAs) in cellular membranes. This process is catalyzed by ROS—particularly hydroxyl radicals and peroxynitrite—under conditions of inflammation, mitochondrial dysfunction, or toxin accumulation [211].

TBARSs are classical but still relevant markers of lipid peroxidation and oxidative stress in CKD. Elevated levels reflect both ongoing renal injury and cardiovascular risk and correlate with inflammation, atherogenic lipid profiles, and nutritional decline [88,212].

As interest in oxidative stress-targeted therapies grows, TBARSs may help stratify risk, monitor response, and refine treatment strategies in CKD populations.

### 4.5. Other Noteworthy CKD-Related Biomarkers: FGF23, Soluble Klotho, and Indoxyl Sulfate

Fibroblast growth factor-23 (FGF23), a phosphate-regulating hormone that rises in early CKD, has been causally linked to left ventricular hypertrophy (LVH) via FGFR4 signaling independent of Klotho, as shown in murine models and corroborated in human studies associating elevated FGF23 with increased LVH and mortality [213,214,215].

Soluble Klotho, a co-receptor for FGF23 with antioxidant and anti-inflammatory effects, declines progressively in CKD; low levels independently predict arterial stiffness and adverse cardiovascular outcomes in human CKD cohorts [216].

Meanwhile, indoxyl sulfate, a gut-derived uremic toxin, accumulates in CKD and stimulates oxidative stress pathways—including NOX activation—promoting endothelial dysfunction, vascular smooth muscle proliferation, and increased vascular disease and mortality risk [217,218,219,220].

## 5. Discussion and Clinical Implications

**Figure 5:**
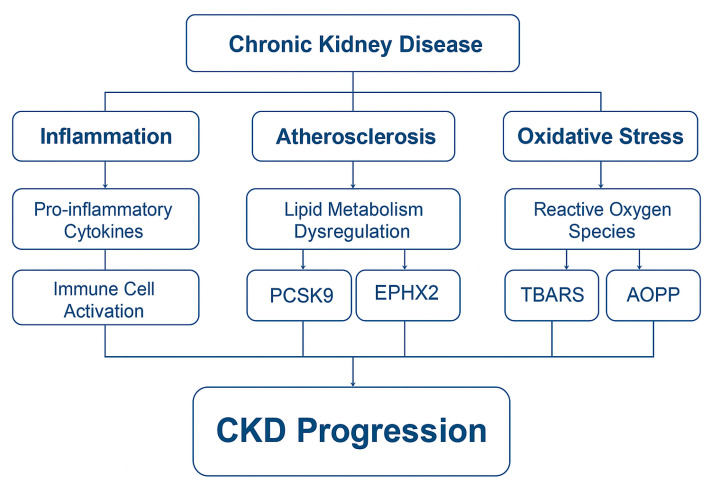
*Key factors in CKD.* (Figure created in BioRender) PCSK9: Proprotein convertase subtilisin/kexin type 9; EPHX2: soluble epoxide hydrolase; AOPPs: advanced oxidation protein products; TBARS: thiobarbituric acid reactive substances.

CKD is a progressive condition characterized by multifactorial pathophysiology (Figure 5) involving oxidative stress, inflammation, lipid dysregulation, and endothelial dysfunction. Despite advances in blood pressure and glycemic control, many patients with CKD—especially those with diabetes—remain at high risk for both kidney failure and cardiovascular events. The integration of emerging biomarkers such as PCSK9, EPHX2, AOPPs, and TBARSs offers a promising paradigm shift in our understanding and management of CKD by targeting and tracking underlying disease-driving mechanisms rather than relying solely on eGFR and albuminuria [221].

### 5.1. Multi-Biomarker Approach in CKD: Complementary Pathways

The biomarkers studied in this review reflect distinct biological domains:

- PCSK9 represents dysregulated lipid metabolism and vascular inflammation;
- EPHX2 reflects endothelial dysfunction and impaired vasoprotective signaling via EET degradation;
- AOPPs and TBARSs quantify oxidative damage to proteins and lipids, respectively, and reflect both uremic toxicity and immune activation.

Rather than functioning in isolation, these molecules interact within a pathophysiological network. For example, PCSK9 not only regulates LDL receptor turnover but also enhances TLR4-mediated inflammation, similar to AOPPs’ interaction with RAGE. TBARSs, as indicators of lipid peroxidation, overlap with EPHX2-induced vascular injury through ROS amplification [195,222,223].

Recent 2025 studies reinforce this systems-level understanding. Liu et al. [129] demonstrated that elevated PCSK9 independently predicts CKD progression in T2DM, highlighting its relevance beyond cholesterol control. Similarly, methylation changes in EPHX2 were associated with DKD severity, suggesting an epigenetic layer of regulation that may be amenable to early detection or intervention [193].

### 5.2. Clinical Utility and Risk Stratification

Incorporating these biomarkers into clinical care has several implications:

Prognostic Risk Models

Adding PCSK9, EPHX2, AOPPs, and TBARSs to traditional markers (eGFR, albuminuria, CRP, lipid profile) may significantly improve risk stratification for the following:

- Renal progression, including ESKD risk;
- MACEs;
- All-cause and cardiovascular mortality, particularly in dialysis.

These biomarkers may help identify high-risk patients who would benefit from more aggressive treatment (e.g., SGLT2 inhibitors, PCSK9 inhibitors) or enrollment in clinical trials.

Nutritional and Inflammatory Monitoring

As shown in our research, a higher AC and lower PNI correlated with poor metabolic and inflammatory status in diabetic nephropathy [33].

Therapeutic Response Monitoring

The dynamic nature of these biomarkers might allow them to be used as pharmacodynamic endpoints. For instance:

- PCSK9 levels decrease in response to evolocumab or inclisiran; a 2025 editorial proposed that PCSK9 inhibition may also preserve tubular megalin expression and reduce proteinuria, opening renal therapeutic indications [144];
- AOPPs decline following MPO inhibition, vitamin E, or N-acetylcysteine, while TBARSs respond to omega-3 fatty acids and CoQ10 supplementation [89,224,225,226];
- EPHX2 inhibitors (e.g., GSK2256294) are under development, and methylation profiling may identify responders vs. non-responders, laying the groundwork for precision nephrology [227].

### 5.3. Toward Personalized Nephrology

Ultimately, these emerging biomarkers may facilitate a shift toward personalized nephrology. Instead of treating based solely on albuminuria or eGFR thresholds, clinicians could do the following [228,229]:

- Stratify patients by molecular phenotype;
- Monitor biochemical responses to interventions;
- Select targeted therapies (e.g., PCSK9 or EPHX2 inhibitors) for the right patients;
- Detect residual cardiovascular or inflammatory risk not captured by traditional markers;
- In parallel, possibly use these biomarkers to help uncover novel therapeutic targets, such as RAGE antagonists for AOPP-mediated damage or NOX4 inhibitors for TBARS-driven lipid injury.

### 5.4. Prognostic Value Beyond Traditional Markers

For biomarkers to influence clinical practice, they must demonstrate prognostic value over traditional measures such as eGFR and albuminuria. Evaluating their associations with CKD progression and stage-specific outcomes provides critical insight into their translational potential. Recent studies have examined whether PCSK9 and AOPPs, beyond their mechanistic roles, can serve as predictors of renal decline and complications.

Recent clinical data indicate that plasma PCSK9 has limited added value for predicting CKD progression beyond standard markers. In moderate CKD cohorts, PCSK9 levels do not correlate with baseline eGFR or albuminuria [131]. For example, the GCKD study found no association between PCSK9 and kidney function indices, and another prospective CKD study showed that an elevated PCSK9 concentration (>220 ng/mL) predicted cardiovascular events but not progression to ESRD (heart failure markers were stronger predictors of renal decline) [131,230]. While one diabetic kidney disease study noted higher PCSK9 levels were linked to worse renal parameters (lower eGFR and higher albuminuria) [231], overall PCSK9 has not demonstrated robust independent prognostic power for CKD progression when traditional risk factors (eGFR, proteinuria) are accounted for [131,230].

In contrast, AOPPs show a stronger association with CKD severity and outcomes. AOPP levels rise progressively with declining renal function, reaching ~3-fold higher than normal in advanced CKD and peaking in dialysis patients [92]. Accumulation of AOPPs is common in CKD and is considered an independent risk factor for oxidative stress-related complications (e.g., cardiovascular events) in this population [195]. Notably, elevated plasma AOPPs have been linked to faster CKD progression: in a prospective IgA nephropathy cohort, a high AOPP level in early disease was one of the most potent independent predictors of poor renal outcome (progression to dialysis). Similarly, AOPP levels correlated with greater proteinuria and more rapid eGFR decline on follow-up [232,233]. These findings suggest that AOPPs correlate with CKD stage and add prognostic information for disease progression beyond eGFR and albuminuria, supporting their role as biomarkers (and potential mediators) of CKD progression [195].

## 6. Limitations and Future Directions

While the integration of emerging biomarkers such as PCSK9, EPHX2, AOPPs, and TBARSs holds immense promise in advancing the precision diagnosis and management of CKD, several critical limitations must be addressed before their widespread clinical application.

### 6.1. Analytical and Methodological Challenges

A major barrier to implementation is assay variability and lack of standardization. TBARS and AOPP assays, despite their frequent use, often lack specificity and exhibit inter-laboratory variability due to nonspecific reactions with structurally similar compounds [87,89]. Furthermore, commercial assays for PCSK9 and EPHX2 are not routinely calibrated for CKD populations, and reference intervals across different stages of kidney disease remain undefined [34,72].

### 6.2. Limited Longitudinal and Interventional Data

Much of the current evidence is derived from cross-sectional or observational studies, limiting conclusions about causality and predictive value. For example, while Liu et al. [129] demonstrated that elevated PCSK9 levels independently predict CKD progression in patients with type 2 diabetes, similar high-quality longitudinal data are lacking for AOPPs and TBARSs. Interventional studies targeting these pathways (e.g., EPHX2 inhibition or antioxidant therapy) remain in early experimental stages or have produced inconclusive clinical outcomes.

### 6.3. Clinical Implementation Barriers

Although the proposed integration of biomarkers with clinical indices (e.g., PNI, AC) offers potential in refining risk stratification, their incorporation into real-world practice is hampered by the absence of validated algorithms, lack of clinical consensus, and limited cost-effectiveness analysis. Moreover, the incremental benefit of these biomarkers over traditional indicators such as eGFR, albuminuria, and CRP must be demonstrated in multicenter validation studies with diverse patient populations [6,49].

### 6.4. Future Research Directions

Key areas for future investigation from our perspective include the following:

- Standardization of assays for oxidative and lipid-associated biomarkers in CKD;
- Prospective, multi-ethnic, and adequately powered cohort studies;
- Interventional trials testing biomarker-guided therapy, including PCSK9 and EPHX2 inhibitors;
- Integration of multi-omics approaches with machine learning to personalize CKD management;
- Development of regulatory pathways and clinical practice guidelines recognizing these markers. Recent advances in epigenetics also open new possibilities. A study by Gao et al. [72] reported EPHX2 methylation patterns associated with diabetic kidney disease severity, suggesting that epigenetic modifications may serve as both diagnostic tools and therapeutic targets.

Addressing these limitations is essential for transitioning these molecular insights from research settings into routine nephrology practice. Their successful integration would mark a significant step toward predictive, pathway-based, and personalized medicine in CKD.

## 7. Conclusions

**Figure 6:**
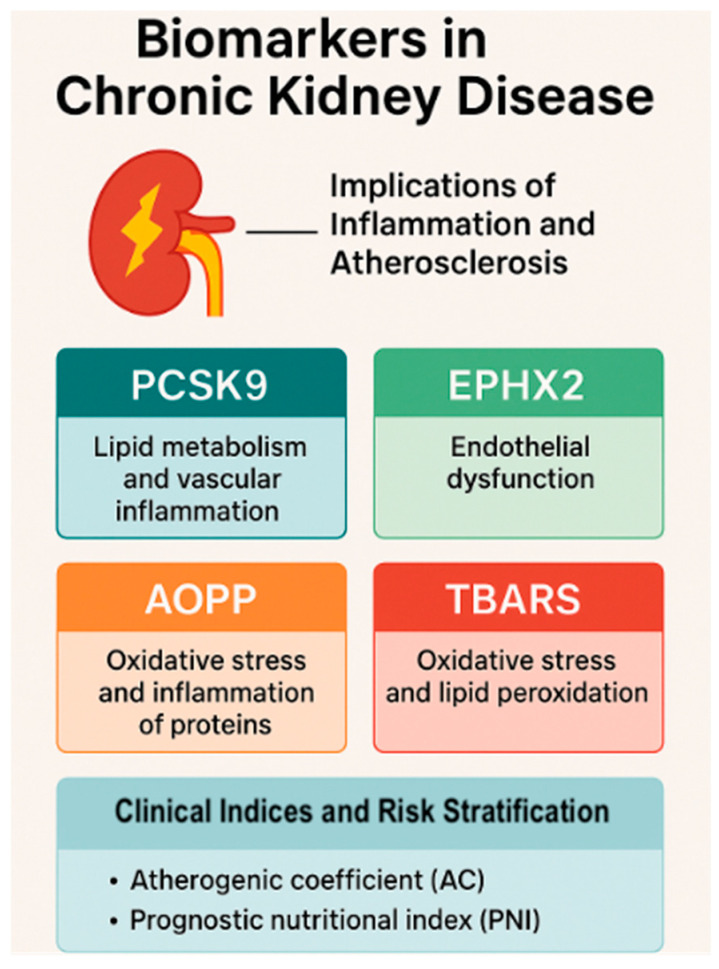
*Biomarkers and clinical indices in CKD.* (Figure created in BioRender) PCSK9: Proprotein convertase subtilisin/kexin type 9; EPHX2: soluble epoxide hydrolase; AOPPs: advanced oxidation protein products; TBARS: thiobarbituric acid reactive substances.

Translating the roles of PCSK9, EPHX2, AOPPs, and TBARSs from bench to bedside offers new hope for slowing CKD progression and reducing cardiovascular burden. Future trials must not only test therapeutic agents targeting these pathways, but also establish standardized, reproducible assays and validate these markers in diverse patient populations. The integration of molecular biomarkers into routine nephrology practice could revolutionize the field, shifting from reactive management to predictive, personalized, and pathway-based intervention. Figure 6 shows a synthesis of integration of serum markers and clinical indices.

While emerging biomarkers such as PCSK9, EPHX2, AOPPs, and TBARSs offer exciting prospects for improving the diagnosis, monitoring, and treatment of CKD, several limitations must be addressed to realize their full clinical potential.

First, analytical variability remains a major barrier. Assays for TBARSs and AOPPs suffer from lack of standardization and limited specificity, which may confound interpretation across studies or patient populations. Similarly, PCSK9 and EPHX2 assays are not yet widely validated for use in nephrology-focused cohorts, and normative reference ranges in CKD remain undefined.

Second, most of the current evidence stems from cross-sectional or small-scale observational studies. Although some recent 2025 studies have begun to establish longitudinal associations—such as PCSK9 predicting CKD progression in diabetes—large, multicenter trials are needed to validate the prognostic utility of these biomarkers and their incremental value beyond traditional markers like eGFR and albuminuria.

Future research should prioritize the following:

- Standardizing assays for oxidative and inflammatory biomarkers;
- Conducting prospective, diverse, and adequately powered cohort studies;
- Integrating multi-omics and machine learning models for personalized prediction;
- Exploring biomarker-guided interventional trials targeting lipid metabolism, oxidative stress, and endothelial dysfunction.

## Figures and Tables

**Table 1:** Comparative summary of PCSK9, EPHX2, AOPPs, TBARSs.

Biomarker	Main Role	CKD Relevance	Pathway	Clinical Utility
PCSK9	LDL metabolism, inflammation	↑ in diabetic nephropathy; predicts MACEs	TLR4/NF-κB	Lipid + inflammatory risk stratification
EPHX2	EET degradation, endothelial injury	Correlates with proteinuria, fibrosis	EET/NO signaling	Renal progression marker; targetable
AOPP	Protein oxidation, immune activation	High in dialysis; predicts malnutrition, CV risk	RAGE, NOX	Oxidative–inflammatory nexus marker
TBARS	Lipid peroxidation	Tracks lipid damage, vascular risk	MDA-DNA/protein adducts	Cardiovascular outcome predictor

MACEs: major adverse cardiovascular events; CKD: Chronic kidney disease; PCSK9: Proprotein convertase subtilisin/kexin type 9; EPHX2: solu-ble epoxide hydrolase; AOPPs: advanced oxidation protein products; TBARS: thiobarbituric acid reactive substances; CV: cardiovascular; LDL: Low-Density Lipoprotein; EET/NO: Epoxyeicosatrienoic acid/nitric oxide; TLR4/NF-κB: Toll-like receptor 4/nuclear factor-κB; RAGE: receptor for advanced glycation end products; NOX: NADPH oxidase. ↑ = activate.

## Data Availability

No new data were created or analyzed in this study.
